# Sex and gender considerations in transplantation research: protocol for a scoping review

**DOI:** 10.1186/s13643-017-0578-4

**Published:** 2017-09-08

**Authors:** Claudie Laprise, Vikas Srinivasan Sridhar, Lori West, Bethany Foster, Louise Pilote, Ruth Sapir-Pichhadze

**Affiliations:** 1 Division of Cancer Epidemiology, McGill University, Montreal, Canada; 2 Division of Oral Health and Society, Faculty of Dentistry, McGill University, Montreal, Canada; 3 Division of General Internal Medicine, McGill University Health Centre Research Institute, Montreal, Canada; 4 Canadian National Transplant Research Program, Edmonton, Canada; 5 Alberta Transplant Institute, University of Alberta, Edmonton, Canada; 6 Department of Pediatrics, Montreal Children’s Hospital of the McGill University Health Centre, McGill University, Montreal, Canada; 7 Division of Clinical Epidemiology, McGill University Health Centre, Research Institute, Montreal, Canada; 8 Division of Nephrology, Department of Medicine, McGill University Health Centre, Montreal, Canada; 9 Metabolic Disorders and Complications, Research Institute of McGill University Health Centre, Montreal, Canada; 10 Multi-Organ Transplant Program, Royal Victoria Hospital, McGill University Health Centre, Montreal, Canada; 11 Centre for Outcomes Research and Evaluation, Research Institute of the McGill University Health Centre, 5252 boul de Maisonneuve, Office 3E.13, Montréal, QC H4A 3S5 Canada

**Keywords:** Sex, Gender, Transplantation, Scoping review

## Abstract

**Background:**

Despite the growing appreciation of the importance of sex and gender considerations in transplantation research, there is currently no framework or good practice guidelines for the appropriate handling of sex and gender issues in human allotransplantation research.

**Methods:**

We will conduct a scoping review to synthesize the evidence on how matters of sex and gender have been handled in human allotransplantation research. We will survey the literature discussing sex and gender in relation to transplantation, including adult and pediatric patients, hematopoietic and solid organ transplant recipients as well as organ donors. We will search MEDLINE and Embase for literature discussing sex and gender in relation to transplantation. Two reviewers will independently evaluate the eligibility of all identified titles and abstracts for inclusion in the full text review, as well as data extraction. Descriptive data and information on how sex and gender have been considered in human transplantation research will be reported.

**Discussion:**

This scoping review will be an important stepping stone towards the development of good practice guidelines on study design and analysis considerations when handling sex and gender issues in human transplantation research. This scoping review can also help identify methodological issues that restrict the translation of transplantation research findings into clinical practice related to underestimation of sex/gender differences. This review will ultimately identify major gaps, inform donor-recipient selection, guide personalized interventions, and prioritize research recommendations in human transplantation research.

**Electronic supplementary material:**

The online version of this article (10.1186/s13643-017-0578-4) contains supplementary material, which is available to authorized users.

## Background

Sex and gender differences in medicine may contribute to disparities in disease incidence, health care system utilization, and general health outcomes. Sex represents a biological characteristic of an individual, while gender refers to the array of socially constructed roles, attitudes, personality traits, and behaviors [1]. These concepts may be conceptually distinct, but also influence and interact with each other [2].

Sex- and gender-based analyses (SGBA) offer a systematic approach to examine the impact of sex and/or gender on population health-related outcomes [3]. SGBA have been implemented in several specialties [4, 5] and have been increasingly recognized as crucial to the development of comprehensive evidence that will ultimately lead to guidelines and policies [6]. Sex and gender are generally understudied in research, and their definitions are often inappropriately interchanged. Many studies incorrectly assume sex and gender neutrality and fail to provide separate analyses based on sex [7]. The failure to consider sex and gender in study design and/or analysis may compromise the validity and generalizability of research findings and affect translation into clinical practice [7, 8]. Greater awareness of patients’ sex and gender will help inform personalized interventions, identify variations of care, and compare effectiveness of therapies.

In human allotransplantation, there is exchange of tissue and/or cells between donors and recipients. Health outcomes, patient experience, and health care costs related to transplantation may vary by transplant candidates’, recipients’, and donors’ sex and/or gender. For example, immune suppression metabolism may vary by sex. Transplant outcomes also vary by donor-recipient sex mismatch. However, the mechanisms leading to sex and gender disparities continue to be debated. Moreover, a marked disparity is observed in access to transplantation by sex. While this disparity may be related to greater immune sensitization in women vs. men from pregnancies, this may also be attributed to a tendency to forgo transplantation, or inability to adhere to appointment or treatment schedules, because of gender role pressure.

Despite the growing appreciation of the importance of sex and gender considerations in research in general, and in transplantation research in particular, there is currently no framework or good practice guidelines for the appropriate handling of sex and gender issues in human allotransplantation research. We will review the available transplantation literature where sex and gender issues were deemed sufficiently central in the manuscript to warrant their mentioning to achieve the following long-term goals: outline personalized strategies to improve transplant outcomes, identify variations in care, ensure equity, inform donor-recipient selection and compatibility evaluation, orient future transplantation research, and inform good practice guidelines for handling sex and gender issues in human allotransplantation research.

## Research objectives

The overall objective of our scoping review is to comprehensively capture how matters of sex and gender have been considered in human allotransplantation research to date. Specifically, this review aims to (1) assess the published literature on the correct application of “sex” and “gender” concepts, (2) ascertain whether (and how) “sex” and “gender” were considered at the stages of study design and analysis, (3) identify the key outcomes for which effects of sex and gender variables were previously considered, and (4) disseminate our research findings. Our scoping review will adhere to the PRISMA-P checklist (Additional file 1).

## Methods

### Scoping review approach

To synthetize the evidence on how matters of sex and gender were handled in human allotransplantation research, we will conduct a scoping review. Scoping reviews are useful for mapping the literature particularly when there is a large body of work that exhibits a large, complex, or heterogeneous nature. While scoping reviews may be undertaken to summarize and disseminate research findings, to identify evidence gaps, to specify policy or practice recommendations, and to make recommendations for the future research, they are primarily used to clarify working definitions and conceptual boundaries of a topic or field and consider various study designs. Unlike systematic reviews, scoping reviews typically do not include a formal quality (risk of bias) appraisal [9–11]. The scoping review approach will allow us to explore the broad topic of sex and gender in relation to transplantation, including adult and pediatric patients, hematopoietic and solid organ transplant recipients, as well as organ donors. Our protocol development follows the framework outlined by Arksey and O’Mally [10] as well as Levac et al. [12], which consists of the (1) identification of the research question, (2) identification of all relevant studies, (3) selection of studies, (4) data abstraction, and (5) summary and reporting of results. Scoping reviews are not eligible for inclusion in PROSPERO. Consequently, a PROSPERO registration number is not provided.

### Search strategy

The electronic database search strategy was developed in consultation with an information specialist. Text words and relevant indexing were used to identify articles discussing sex and/or gender issues in transplantation (hematopoietic and solid organ) and in donation. MEDLINE and Embase were searched using the following keywords: sex difference, sex, or gender (with characteristics, factor, imbalance, issue, specific) in combination with the keywords tissue, cell transplantation (tissue, cell or hematopoietic transplant, donors, donation), or organ (cardiac, heart, hematopoietic, hepatic, kidney, liver, lung, organ, pancreas, pulmonic, renal, donor, donation) transplantation. The search strategy presented in Table 1 yielded 6083 unique references.

**Table 1:** Search strategies in Medline and Embase electronic databases for sex and gender in transplantation

Ovid Medline	Ovid Embase
1. Sex Factors 2. Sex Characteristics 3. ((sex or gender) adj3 (characteristic* or factor* or imbalance* or issue* or specific*)).tw,kf. 4. ((sex or gender) adj3 differenc*).ti,kf. 5. ((sex or gender) adj3 differenc*).ab. /freq = 2 6. or/1-5 7. exp cell transplantation 8. ((cell* or hematopoietic) adj3 transplant*).tw,kf. 9. 7 or 8 10. 6 and 9 11. exp. organ transplantation 12. ((cardiac or heart or hepatic or kidney* or liver or lung or organ or pancrea* or pulmon* or renal) adj3 (graft* or transplant*)).tw,kf. 13. 11 or 12 14. 6 and 13 15. Living Donors 16. Tissue Donors 17. ((cell* or cardiac or heart or hematopoietic or hepatic or kidney* or liver or lung or organ or pancrea* or pulmon* or renal) adj3 (donor* or donation*)).tw,kf. 18. or/15-17 19. 6 and 18 20. 10 or 14 or 19	1. Sex difference 2. ((sex or gender) adj3 (characteristic* or factor* or imbalance* or issue* or specific*)).tw,kw. 3. ((sex or gender) adj3 differenc*).ti,kw. 4. ((sex or gender) adj3 differenc*).ab. /freq = 2 5. or/1-4 6. exp cell transplantation 7. ((cell* or hematopoietic) adj3 transplant*).tw,kw. 8. 6 or 7 9. 5 and 8 10. exp. organ transplantation 11. ((cardiac or heart or hepatic or kidney* or liver or lung or organ or pancrea* or pulmon* or renal) adj3 (graft* or transplant*)).tw,kw. 12. 10 or 11 ( 13. 5 and 12 14. living donor 15. kidney donor 16. organ donor 17. unrelated donors 18. ((cell* or cardiac or heart or hematopoietic or hepatic or kidney* or liver or lung or organ or pancrea* or pulmon* or renal) adj3 (donor* or donation*)).tw,kw. 19. or/14-18 20. 5 and 19 21. 9 or 13 or 20

**Table 2:** Summary of planned data extraction from full-text articles selected for inclusion in the scoping review

Study characteristics	Extracted data
General information	• Authors, country of origin, title, year of publication
Study design	• Systematic review/meta-analysis, interventional (RCT), observational (cohort, case-control, cross sectional/survey) • For longitudinal studies, the beginning and end year of the study
Study type	• Descriptive and/or analytical
Population characteristics	• Selection criteria (inclusion/exclusion) • Adult, pediatric • Waitlisted transplant candidates, transplant recipients and/or donors
Sample size	• Number of participants (overall, by sex/gender)
Type of organ under study	• Stem cells, tissues, and/or solid organs
Exposure, outcome, and covariates	• Exposure: health risk factors/predictors • The main health outcome under study (e.g., patient/graft survival, pharmacokinetics/pharmacodynamics, health services access, utilization, interaction with the healthcare system, cost) • Covariates ° Sex related covariates: anatomy (reproductive organ, proportion of fat and muscle), biological variables (hormones, genetic profile, gene expression), physiological variables (blood and serologic parameters) ° Gender-related covariates: gender roles (housework, child care), gender identity (personality traits), gender relationships (social support), institutionalized gender (education level, profession, personal income), behavioral and cultural variables (smoking, drinking, occupation
Research theme	• Biomedical, clinical, health system/services, population health
Sex and gender consideration	
Appropriate application of definitions	• Yes • No (Sex and gender used interchangeably and erroneously)
Inclusion in the primary research question/objective/hypothesis	• Yes • No
Consideration in study design	• Are both sexes considered? (y/n) • Were only men or women included? (y/n) • Was an explanation for sex/gender exclusion/inclusion provided (e.g., not necessary, not feasible)? (y/n)
Consideration in analysis	• How was sex considered in the analyses? ° Descriptive (Table 1) ° Primary exposure (effect size) ° Confounder (included in multivariable models, restriction (sensitivity and/or subgroup analyses) or effect measure modifier (included in interaction terms followed by stratification of the main effect by sex if the interaction term is statistically significant) • How was gender considered in the analyses? ° Gender was accounted for by considering sex-related associations ° Gender story was sought by applying second-level disaggregation by sex ° Gender story was sought by using gender-related variables ° Gender index/score was created to analyze gender independently of sex ▪ Summing up different variables to create a score ▪ Regression variables to predict male/female sex ▪ Factor analysis to capture underlying gender-based constructs ▪ Create a ranking based on the male/female distribution to a single variable
Consideration in study results reporting	• Were analyses by sex and/or gender clearly presented in the results section, tables, and/or figures? ° Levels of sex and gender integration: ▪ Gender unequal ▪ Gender blind ▪ Gender sensitive ▪ Gender specific ▪ Gender transformative
Consideration in the discussion, conclusion, and/or recommendations	• Statements, limitations pertinent to sex and gender research

### Study selection

Studies will be eligible for inclusion if they satisfy the following criteria: (1) the target population consists of humans across the age continuum undergoing stem cell (e.g., allogenic or mesenchymal), tissue (e.g., allogenic skin, bone, and cornea grafts), or solid organ transplantation in the capacity of transplant candidates, recipients, and donors; (2) sex and/or gender are mentioned in the title or abstract of the manuscript; (3) the manuscript reports original research, written in English, and published between January 1, 1946 and October 17, 2016; (4) the study design includes randomized control trials, observational studies, case series with at least 20 participants, registry/population report, validation survey, and method comparison with at least 20 participants.

Studies will be excluded if they (1) do not discuss allotransplantation (e.g. ventricular assist devices that do not serve as bridges to transplantation, vein allografts for cardiac bypass, homografts (heart valves), transfusion/donation of blood products, tumor/cancer transplants, embryo oocytes or sperm donation, brain or body donation for research, and autologous grafts), (2) do not mention sex or gender in the title and/or abstract, (3) are not original studies (letters, editorials, news, replies, comments), or (4) are conference abstracts.

Two reviewers will independently evaluate the eligibility of all identified titles and abstracts for inclusion in the full-text review. Disagreements will be resolved by consensus or by a third reviewer. Full-text articles will be evaluated for inclusion by two independent reviewers using similar inclusion and exclusion criteria (Table 1). All included studies will be synthetized and reported in a separate appendix.

### Data extraction, analysis, and synthesis

Data extraction from selected full-text manuscripts will be done using Covidence, a systematic review software developed in partnership with the Cochrane Collaboration, and will comprise of two steps. First, descriptive data will be extracted into a form, which will include the variables appearing in Table 2 (general information on the study, population, setting, study design and type, sample size, transplanted cells/tissues/organs, main outcomes, and sex- and gender-related covariates). Second, for each selected study, to evaluate how sex and gender have been considered in human transplantation research to date, two reviewers will independently extract data with disagreements when answering the following questions resolved by consensus or by a third reviewer:

1. Were the concepts of sex and gender used appropriately, or were they used interchangeably and/or erroneously?
2. Were sex and/or gender mentioned in the primary research question?
3. Were sex and/or gender considered in the study design (e.g., (i) participants selected by sex or gender or study sample stratified by sex), and was a reason specified (not necessary or not feasible)?
4. Were sex and gender considered in the statistical analysis (i.e., considered as (i) covariates in multivariable models, (ii) as effect measure modifiers and/or included as interaction terms in multivariable models, or (iii) were there sensitivity or subgroup analyses determined by participants’ sex or gender)?
5. How were gender related variables measured (e.g., gender roles, identity, relations)?
6. Were analyses by sex and/or gender reported in the results (i.e., SGBA results reported separately and presented in tables and figures)?

We will present descriptive statistics of the study characteristics outlined in Table 2 (e.g., by study era, country, etc.). We will summarize the proportion of manuscripts applying the term “gender” correctly as well as whether and how gender-related variables were measured and reported in the manuscript. Finally, we will provide narrative synthesis on how sex and gender were handled at the level of study design and analysis.

## Discussion

**Fig. 1:**
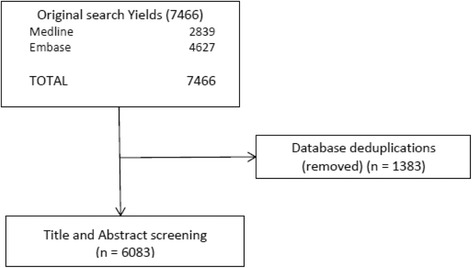
Preliminary study research results for sex and gender in transplantation

To our knowledge, this scoping review will be the first evaluating how sex and gender have been handled in human allotransplantation research. This review will comprehensively inform the various key players in transplantation ranging from donors, through transplant candidates and recipients, to transplant physicians and coordinators. A few foreseen limitations must be noted, however. Our selection criteria may decrease the likelihood of detecting articles discussing gender/sex concepts in transplantation when sex and gender are mentioned in the text but not in the title or abstract. Articles discussing gender-related variables without specifically using the term “gender” may not be captured. We might also exclude smaller case series, potentially leading to under-representation of less frequently transplanted cells/tissues/organs. Despite these limitations, we believe that the findings of our scoping review will be an important stepping stone towards the development of good practice guidelines on study design and analysis considerations when handling sex and gender issues in human transplantation research (Fig. 1). This scoping review will also help identify methodological issues that restrict the translation of transplantation research findings into clinical practice related to underestimation of sex/gender differences. This ultimately will serve to identify major gaps in the literature, inform donor-recipient selection, guide personalized interventions, and prioritize research recommendations in human transplantation research. Ultimately, these efforts are expected to inform personalized management that can improve health outcomes, patient experience, and health expenditure in transplant recipients.

## Additional file

Additional file 1: PRISMA-P 2015 Checklist. (DOCX 30 kb)

## Abbreviations

SGBA: Sex- and gender-based analyses
